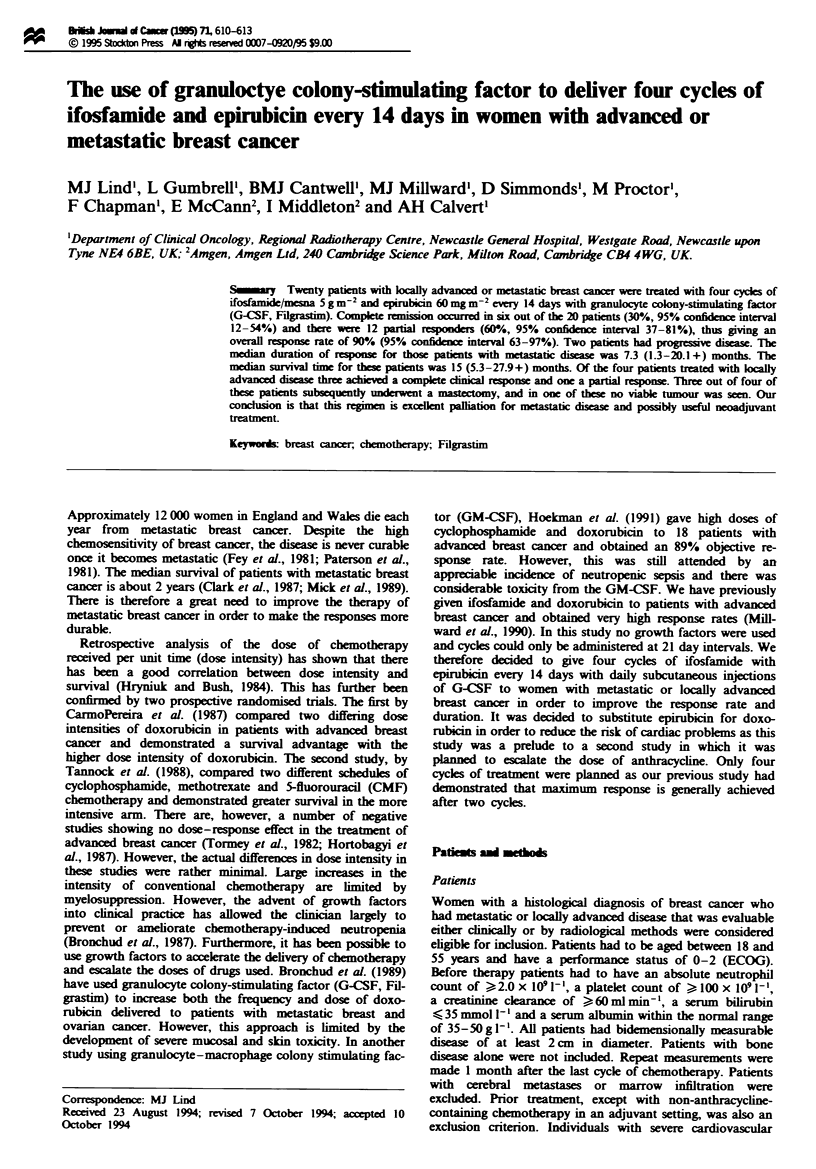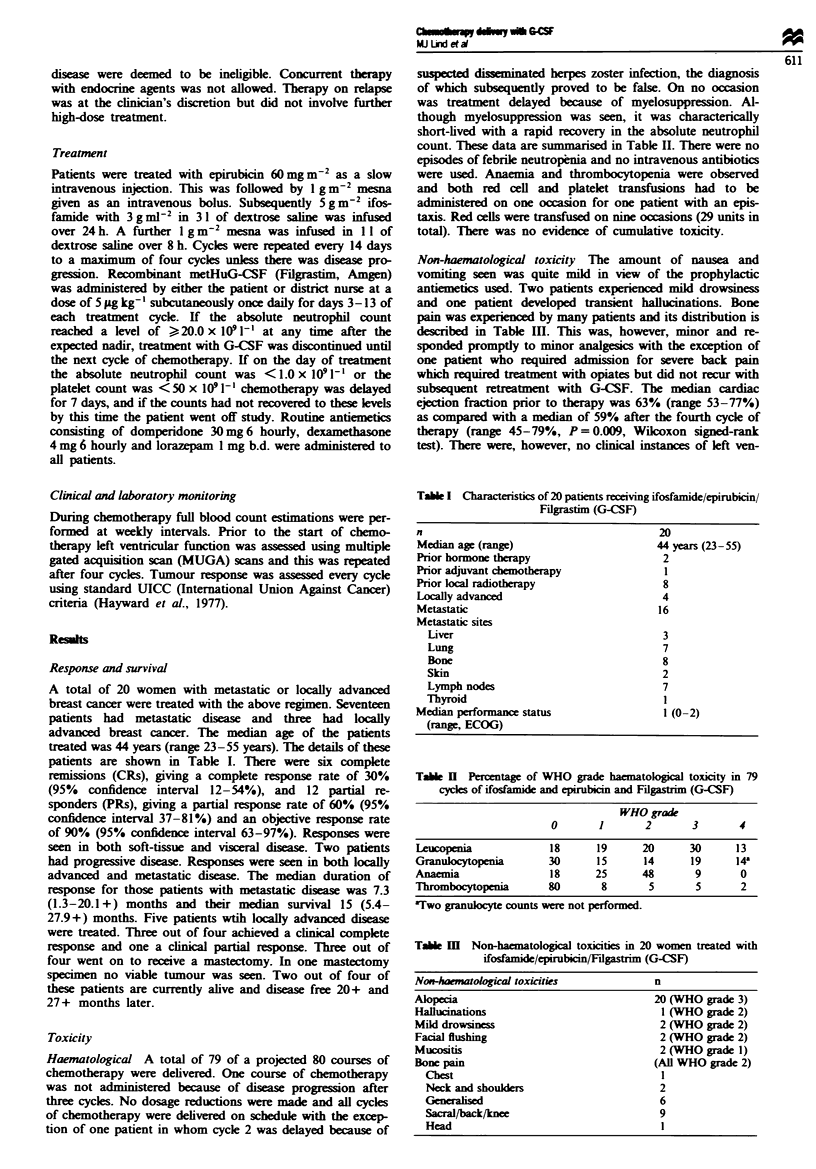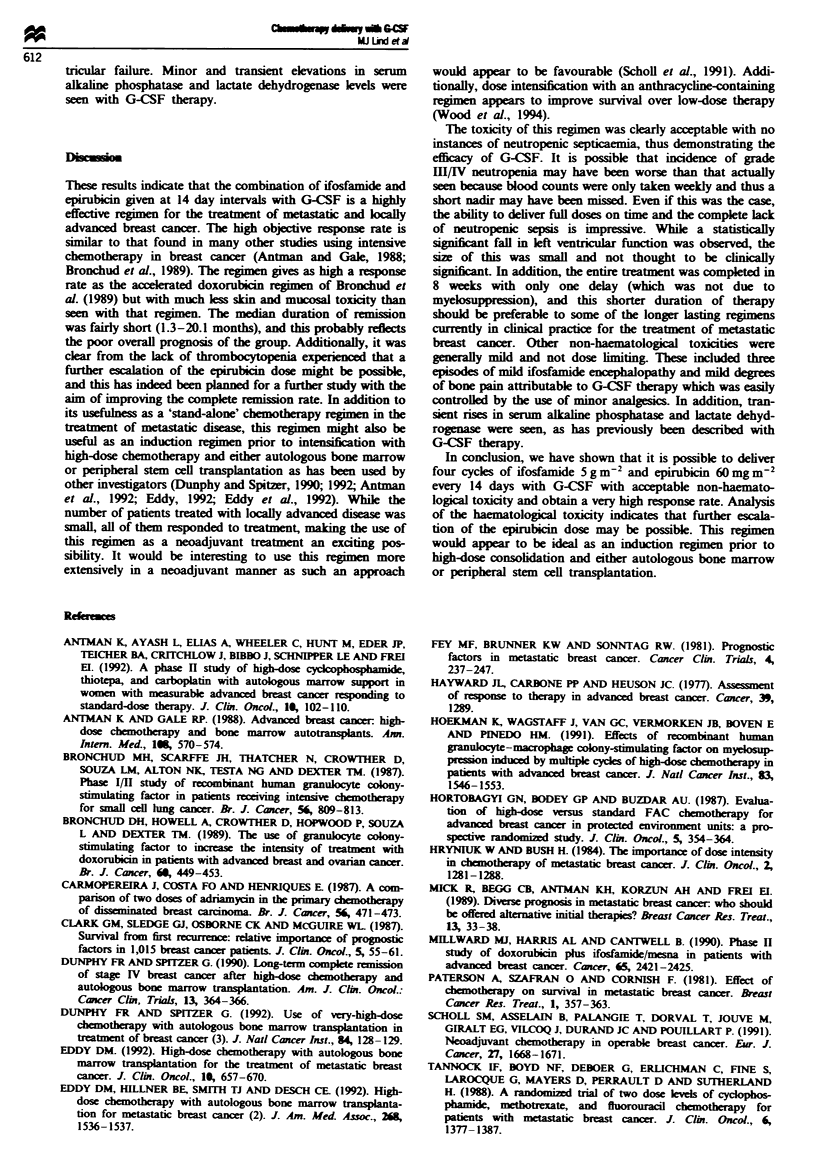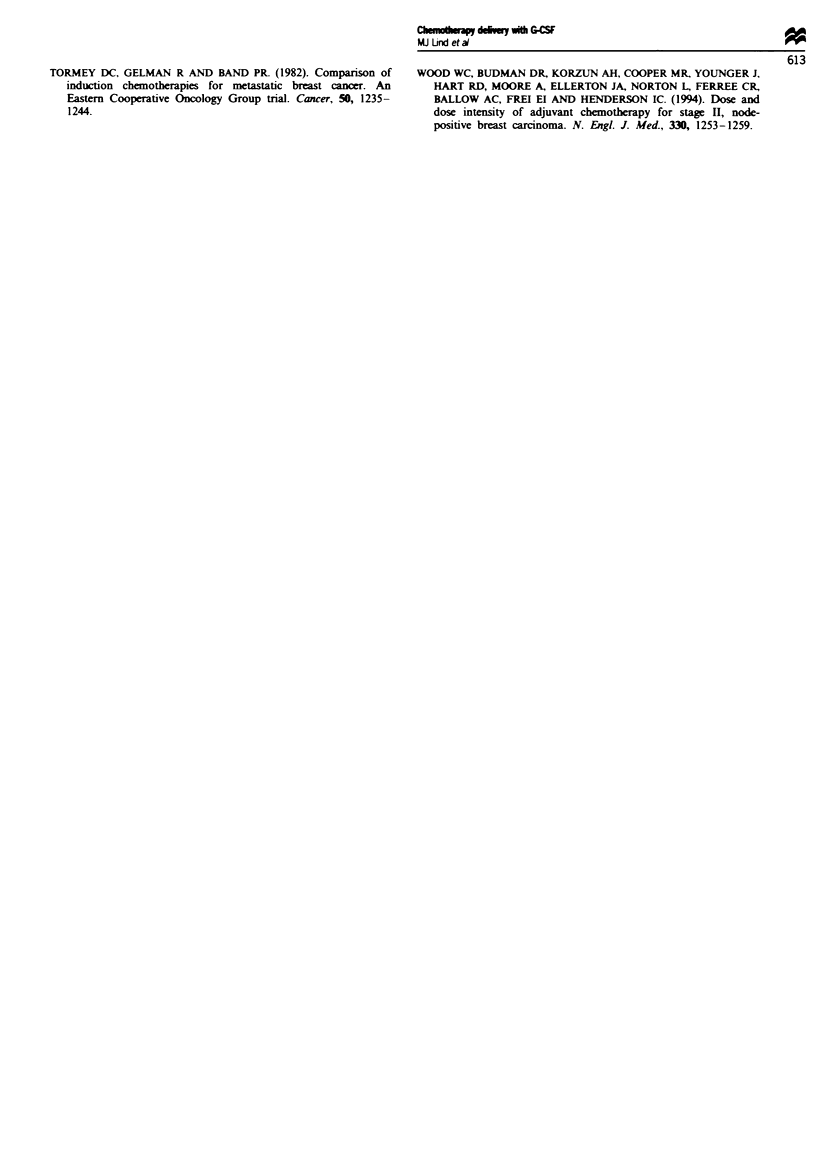# The use of granulocyte colony-stimulating factor to deliver four cycles of ifosfamide and epirubicin every 14 days in women with advanced or metastatic breast cancer.

**DOI:** 10.1038/bjc.1995.118

**Published:** 1995-03

**Authors:** M. J. Lind, L. Gumbrell, B. M. Cantwell, M. J. Millward, D. Simmonds, M. Proctor, F. Chapman, E. McCann, I. Middleton, A. H. Calvert

**Affiliations:** Department of Clinical Oncology, Newcastle General Hospital, Newcastle upon Tyne, UK.

## Abstract

Twenty patients with locally advanced or metastatic breast cancer were treated with four cycles of ifosfamide/mesna 5 g m-2 and epirubicin 60 mg m-2 every 14 days with granulocyte colony-stimulating factor (G-CSF, Filgrastim). Complete remission occurred in six out of the 20 patients (30%, 95% confidence interval 12-54%) and there were 12 partial responders (60%, 95% confidence interval 37-81%), thus giving an overall response rate of 90% (95% confidence interval 63-97%). Two patients had progressive disease. The median duration of response for those patients with metastatic disease was 7.3 (1.3-20.1+) months. The median survival time for these patients was 15 (5.3-27.9+) months. Of the four patients treated with locally advanced disease three achieved a complete clinical response and one a partial response. Three out of four of these patients subsequently underwent a mastectomy, and in one of these no viable tumour was seen. Our conclusion is that this regimen is excellent palliation for metastatic disease and possibly useful neoadjuvant treatment.


© 1995 Stockton Press All rights reserved 0007–0920/95 $9.00

### The use of granuloctye colony-stimulating factor to deliver four cycles of ifosfamide and epirubicin every 14 days in women with advanced or metastatic breast cancer

MJ Lind[1], L Gumbrell[1], BMJ Cantwell[1], MJ Millward[1], D Simmonds[1], M Proctor[1], F Chapman[1], E McCann[2], I Middleton[2] and AH Calvert[1]

[1]*Department of Clinical Oncology, Regional Radiotherapy Centre, Newcastle General Hospital, Westgate Road, Newcastle upon Tyne NE4 6BE, UK;* [2]*Amgen, Amgen Ltd, 240 Cambridge Science Park, Milton Road, Cambridge CB4 4WG, UK.*

**Summary** Twenty patients with locally advanced or metastatic breast cancer were treated with four cycles of ifosfamide/mesna 5 g $m^{-2}$ and epirubicin 60 mg $m^{-2}$ every 14 days with granulocyte colony-stimulating factor (G-CSF, Filgrastim). Complete remission occurred in six out of the 20 patients (30%, 95% confidence interval 12–54%) and there were 12 partial responders (60%, 95% confidence interval 37–81%), thus giving an overall response rate of 90% (95% confidence interval 63–97%). Two patients had progressive disease. The median duration of response for those patients with metastatic disease was 7.3 (1.3–20.1+) months. The median survival time for these patients was 15 (5.3–27.9+) months. Of the four patients treated with locally advanced disease three achieved a complete clinical response and one a partial response. Three out of four of these patients subsequently underwent a mastectomy, and in one of these no viable tumour was seen. Our conclusion is that this regimen is excellent palliation for metastatic disease and possibly useful neoadjuvant treatment.

**Keywords**: breast cancer; chemotherapy; Filgrastim

Approximately 12 000 women in England and Wales die each year from metastatic breast cancer. Despite the high chemosensitivity of breast cancer, the disease is never curable once it becomes metastatic (Fey *et al.*, 1981; Paterson *et al.*, 1981). The median survival of patients with metastatic breast cancer is about 2 years (Clark *et al.*, 1987; Mick *et al.*, 1989). There is therefore a great need to improve the therapy of metastatic breast cancer in order to make the responses more durable.

Retrospective analysis of the dose of chemotherapy received per unit time (dose intensity) has shown that there has been a good correlation between dose intensity and survival (Hryniuk and Bush, 1984). This has further been confirmed by two prospective randomised trials. The first by CarmoPereira *et al.* (1987) compared two differing dose intensities of doxorubicin in patients with advanced breast cancer and demonstrated a survival advantage with the higher dose intensity of doxorubicin. The second study, by Tannock *et al.* (1988), compared two different schedules of cyclophosphamide, methotrexate and 5-fluorouracil (CMF) chemotherapy and demonstrated greater survival in the more intensive arm. There are, however, a number of negative studies showing no dose–response effect in the treatment of advanced breast cancer (Tormey *et al.*, 1982; Hortobagyi *et al.*, 1987). However, the actual differences in dose intensity in these studies were rather minimal. Large increases in the intensity of conventional chemotherapy are limited by myelosuppression. However, the advent of growth factors into clinical practice has allowed the clinician largely to prevent or ameliorate chemotherapy-induced neutropenia (Bronchud *et al.*, 1987). Furthermore, it has been possible to use growth factors to accelerate the delivery of chemotherapy and escalate the doses of drugs used. Bronchud *et al.* (1989) have used granulocyte colony-stimulating factor (G-CSF, Filgrastim) to increase both the frequency and dose of doxorubicin delivered to patients with metastatic breast and ovarian cancer. However, this approach is limited by the development of severe mucosal and skin toxicity. In another study using granulocyte–macrophage colony stimulating factor (GM-CSF), Hoekman *et al.* (1991) gave high doses of cyclophosphamide and doxorubicin to 18 patients with advanced breast cancer and obtained an 89% objective response rate. However, this was still attended by an appreciable incidence of neutropenic sepsis and there was considerable toxicity from the GM-CSF. We have previously given ifosfamide and doxorubicin to patients with advanced breast cancer and obtained very high response rates (Millward *et al.*, 1990). In this study no growth factors were used and cycles could only be administered at 21 day intervals. We therefore decided to give four cycles of ifosfamide with epirubicin every 14 days with daily subcutaneous injections of G-CSF to women with metastatic or locally advanced breast cancer in order to improve the response rate and duration. It was decided to substitute epirubicin for doxorubicin in order to reduce the risk of cardiac problems as this study was a prelude to a second study in which it was planned to escalate the dose of anthracycline. Only four cycles of treatment were planned as our previous study had demonstrated that maximum response is generally achieved after two cycles.

### Patients and methods

### *Patients*

Women with a histological diagnosis of breast cancer who had metastatic or locally advanced disease that was evaluable either clinically or by radiological methods were considered eligible for inclusion. Patients had to be aged between 18 and 55 years and have a performance status of 0–2 (ECOG). Before therapy patients had to have an absolute neutrophil count of $\geqslant 2.0 \times 10^9 l^{-1}$, a platelet count of $\geqslant 100 \times 10^9 l^{-1}$, a creatinine clearance of $\geqslant 60$ ml $min^{-1}$, a serum bilirubin $\leqslant 35$ mmol $l^{-1}$ and a serum albumin within the normal range of 35–50 g $l^{-1}$. All patients had bidemensionally measurable disease of at least 2 cm in diameter. Patients with bone disease alone were not included. Repeat measurements were made 1 month after the last cycle of chemotherapy. Patients with cerebral metastases or marrow infiltration were excluded. Prior treatment, except with non-anthracycline-containing chemotherapy in an adjuvant setting, was also an exclusion criterion. Individuals with severe cardiovascular

Correspondence: MJ Lind
Received 23 August 1994; revised 7 October 1994; accepted 10 October 1994

disease were deemed to be ineligible. Concurrent therapy with endocrine agents was not allowed. Therapy on relapse was at the clinician's discretion but did not involve further high-dose treatment.

*Treatment*

Patients were treated with epirubicin 60 mg $m^{-2}$ as a slow intravenous injection. This was followed by 1 g $m^{-2}$ mesna given as an intravenous bolus. Subsequently 5 g $m^{-2}$ ifosfamide with 3 g $ml^{-2}$ in 3 l of dextrose saline was infused over 24 h. A further 1 g $m^{-2}$ mesna was infused in 1 l of dextrose saline over 8 h. Cycles were repeated every 14 days to a maximum of four cycles unless there was disease progression. Recombinant metHuG-CSF (Filgrastim, Amgen) was administered by either the patient or district nurse at a dose of 5 $\mu g\ kg^{-1}$ subcutaneously once daily for days 3–13 of each treatment cycle. If the absolute neutrophil count reached a level of $\geqslant 20.0 \times 10^9 l^{-1}$ at any time after the expected nadir, treatment with G-CSF was discontinued until the next cycle of chemotherapy. If on the day of treatment the absolute neutrophil count was $<1.0 \times 10^9 l^{-1}$ or the platelet count was $<50 \times 10^9 l^{-1}$ chemotherapy was delayed for 7 days, and if the counts had not recovered to these levels by this time the patient went off study. Routine antiemetics consisting of domperidone 30 mg 6 hourly, dexamethasone 4 mg 6 hourly and lorazepam 1 mg b.d. were administered to all patients.

*Clinical and laboratory monitoring*

During chemotherapy full blood count estimations were performed at weekly intervals. Prior to the start of chemotherapy left ventricular function was assessed using multiple gated acquisition scan (MUGA) scans and this was repeated after four cycles. Tumour response was assessed every cycle using standard UICC (International Union Against Cancer) criteria (Hayward *et al.*, 1977).

### Results

*Response and survival*

A total of 20 women with metastatic or locally advanced breast cancer were treated with the above regimen. Seventeen patients had metastatic disease and three had locally advanced breast cancer. The median age of the patients treated was 44 years (range 23–55 years). The details of these patients are shown in Table I. There were six complete remissions (CRs), giving a complete response rate of 30% (95% confidence interval 12–54%), and 12 partial responders (PRs), giving a partial response rate of 60% (95% confidence interval 37–81%) and an objective response rate of 90% (95% confidence interval 63–97%). Responses were seen in both soft-tissue and visceral disease. Two patients had progressive disease. Responses were seen in both locally advanced and metastatic disease. The median duration of response for those patients with metastatic disease was 7.3 (1.3–20.1+) months and their median survival 15 (5.4–27.9+) months. Five patients wtih locally advanced disease were treated. Three out of four achieved a clinical complete response and one a clinical partial response. Three out of four went on to receive a mastectomy. In one mastectomy specimen no viable tumour was seen. Two out of four of these patients are currently alive and disease free 20+ and 27+ months later.

*Toxicity*

*Haematological* A total of 79 of a projected 80 courses of chemotherapy were delivered. One course of chemotherapy was not administered because of disease progression after three cycles. No dosage reductions were made and all cycles of chemotherapy were delivered on schedule with the exception of one patient in whom cycle 2 was delayed because of suspected disseminated herpes zoster infection, the diagnosis of which subsequently proved to be false. On no occasion was treatment delayed because of myelosuppression. Although myelosuppression was seen, it was characterically short-lived with a rapid recovery in the absolute neutrophil count. These data are summarised in Table II. There were no episodes of febrile neutropènia and no intravenous antibiotics were used. Anaemia and thrombocytopenia were observed and both red cell and platelet transfusions had to be administered on one occasion for one patient with an epistaxis. Red cells were transfused on nine occasions (29 units in total). There was no evidence of cumulative toxicity.

*Non-haematological toxicity* The amount of nausea and vomiting seen was quite mild in view of the prophylactic antiemetics used. Two patients experienced mild drowsiness and one patient developed transient hallucinations. Bone pain was experienced by many patients and its distribution is described in Table III. This was, however, minor and responded promptly to minor analgesics with the exception of one patient who required admission for severe back pain which required treatment with opiates but did not recur with subsequent retreatment with G-CSF. The median cardiac ejection fraction prior to therapy was 63% (range 53–77%) as compared with a median of 59% after the fourth cycle of therapy (range 45–79%, $P = 0.009$, Wilcoxon signed-rank test). There were, however, no clinical instances of left ven-

**Table I** Characteristics of 20 patients receiving ifosfamide/epirubicin/Filgrastim (G-CSF)

| | |
|---|---|
| *n* | 20 |
| Median age (range) | 44 years (23–55) |
| Prior hormone therapy | 2 |
| Prior adjuvant chemotherapy | 1 |
| Prior local radiotherapy | 8 |
| Locally advanced | 4 |
| Metastatic | 16 |
| Metastatic sites | |
| Liver | 3 |
| Lung | 7 |
| Bone | 8 |
| Skin | 2 |
| Lymph nodes | 7 |
| Thyroid | 1 |
| Median performance status (range, ECOG) | 1 (0–2) |

**Table II** Percentage of WHO grade haematological toxicity in 79 cycles of ifosfamide and epirubicin and Filgastrim (G-CSF)

| | *WHO grade* | | | | |
|---|---|---|---|---|---|
| | *0* | *1* | *2* | *3* | *4* |
| Leucopenia | 18 | 19 | 20 | 30 | 13 |
| Granulocytopenia | 30 | 15 | 14 | 19 | 14[a] |
| Anaemia | 18 | 25 | 48 | 9 | 0 |
| Thrombocytopenia | 80 | 8 | 5 | 5 | 2 |

[a]Two granulocyte counts were not performed.

**Table III** Non-haematological toxicities in 20 women treated with ifosfamide/epirubicin/Filgastrim (G-CSF)

| *Non-haematological toxicities* | n |
|---|---|
| Alopecia | 20 (WHO grade 3) |
| Hallucinations | 1 (WHO grade 2) |
| Mild drowsiness | 2 (WHO grade 2) |
| Facial flushing | 2 (WHO grade 2) |
| Mucositis | 2 (WHO grade 1) |
| Bone pain | (All WHO grade 2) |
| Chest | 1 |
| Neck and shoulders | 2 |
| Generalised | 6 |
| Sacral/back/knee | 9 |
| Head | 1 |

tricular failure. Minor and transient elevations in serum alkaline phosphatase and lactate dehydrogenase levels were seen with G-CSF therapy.

### Discussion

These results indicate that the combination of ifosfamide and epirubicin given at 14 day intervals with G-CSF is a highly effective regimen for the treatment of metastatic and locally advanced breast cancer. The high objective response rate is similar to that found in many other studies using intensive chemotherapy in breast cancer (Antman and Gale, 1988; Bronchud *et al.*, 1989). The regimen gives as high a response rate as the accelerated doxorubicin regimen of Bronchud *et al.* (1989) but with much less skin and mucosal toxicity than seen with that regimen. The median duration of remission was fairly short (1.3–20.1 months), and this probably reflects the poor overall prognosis of the group. Additionally, it was clear from the lack of thrombocytopenia experienced that a further escalation of the epirubicin dose might be possible, and this has indeed been planned for a further study with the aim of improving the complete remission rate. In addition to its usefulness as a 'stand-alone' chemotherapy regimen in the treatment of metastatic disease, this regimen might also be useful as an induction regimen prior to intensification with high-dose chemotherapy and either autologous bone marrow or peripheral stem cell transplantation as has been used by other investigators (Dunphy and Spitzer, 1990; 1992; Antman *et al.*, 1992; Eddy, 1992; Eddy *et al.*, 1992). While the number of patients treated with locally advanced disease was small, all of them responded to treatment, making the use of this regimen as a neoadjuvant treatment an exciting possibility. It would be interesting to use this regimen more extensively in a neoadjuvant manner as such an approach would appear to be favourable (Scholl *et al.*, 1991). Additionally, dose intensification with an anthracycline-containing regimen appears to improve survival over low-dose therapy (Wood *et al.*, 1994).

The toxicity of this regimen was clearly acceptable with no instances of neutropenic septicaemia, thus demonstrating the efficacy of G-CSF. It is possible that incidence of grade III/IV neutropenia may have been worse than that actually seen because blood counts were only taken weekly and thus a short nadir may have been missed. Even if this was the case, the ability to deliver full doses on time and the complete lack of neutropenic sepsis is impressive. While a statistically significant fall in left ventricular function was observed, the size of this was small and not thought to be clinically significant. In addition, the entire treatment was completed in 8 weeks with only one delay (which was not due to myelosuppression), and this shorter duration of therapy should be preferable to some of the longer lasting regimens currently in clinical practice for the treatment of metastatic breast cancer. Other non-haematological toxicities were generally mild and not dose limiting. These included three episodes of mild ifosfamide encephalopathy and mild degrees of bone pain attributable to G-CSF therapy which was easily controlled by the use of minor analgesics. In addition, transient rises in serum alkaline phosphatase and lactate dehydrogenase were seen, as has previously been described with G-CSF therapy.

In conclusion, we have shown that it is possible to deliver four cycles of ifosfamide $5\ g\ m^{-2}$ and epirubicin $60\ mg\ m^{-2}$ every 14 days with G-CSF with acceptable non-haematological toxicity and obtain a very high response rate. Analysis of the haematological toxicity indicates that further escalation of the epirubicin dose may be possible. This regimen would appear to be ideal as an induction regimen prior to high-dose consolidation and either autologous bone marrow or peripheral stem cell transplantation.

### References

ANTMAN K, AYASH L, ELIAS A, WHEELER C, HUNT M, EDER JP, TEICHER BA, CRITCHLOW J, BIBBO J, SCHNIPPER LE AND FREI EI. (1992). A phase II study of high-dose cyclophosphamide, thiotepa, and carboplatin with autologous marrow support in women with measurable advanced breast cancer responding to standard-dose therapy. *J. Clin. Oncol.*, **10**, 102–110.

ANTMAN K AND GALE RP. (1988). Advanced breast cancer: high-dose chemotherapy and bone marrow autotransplants. *Ann. Intern. Med.*, **108**, 570–574.

BRONCHUD MH, SCARFFE JH, THATCHER N, CROWTHER D, SOUZA LM, ALTON NK, TESTA NG AND DEXTER TM. (1987). Phase I/II study of recombinant human granulocyte colony-stimulating factor in patients receiving intensive chemotherapy for small cell lung cancer. *Br. J. Cancer*, **56**, 809–813.

BRONCHUD DH, HOWELL A, CROWTHER D, HOPWOOD P, SOUZA L AND DEXTER TM. (1989). The use of granulocyte colony-stimulating factor to increase the intensity of treatment with doxorubicin in patients with advanced breast and ovarian cancer. *Br. J. Cancer*, **60**, 449–453.

CARMOPEREIRA J, COSTA FO AND HENRIQUES E. (1987). A comparison of two doses of adriamycin in the primary chemotherapy of disseminated breast carcinoma. *Br. J. Cancer*, **56**, 471–473.

CLARK GM, SLEDGE GJ, OSBORNE CK AND McGUIRE WL. (1987). Survival from first recurrence: relative importance of prognostic factors in 1,015 breast cancer patients. *J. Clin. Oncol.*, **5**, 55–61.

DUNPHY FR AND SPITZER G. (1990). Long-term complete remission of stage IV breast cancer after high-dose chemotherapy and autologous bone marrow transplantation. *Am. J. Clin. Oncol.: Cancer Clin, Trials*, **13**, 364–366.

DUNPHY FR AND SPITZER G. (1992). Use of very-high-dose chemotherapy with autologous bone marrow transplantation in treatment of breast cancer (3). *J. Natl Cancer Inst.*, **84**, 128–129.

EDDY DM. (1992). High-dose chemotherapy with autologous bone marrow transplantation for the treatment of metastatic breast cancer. *J. Clin. Oncol.*, **10**, 657–670.

EDDY DM, HILLNER BE, SMITH TJ AND DESCH CE. (1992). High-dose chemotherapy with autologous bone marrow transplantation for metastatic breast cancer (2). *J. Am. Med. Assoc.*, **268**, 1536–1537.

FEY MF, BRUNNER KW AND SONNTAG RW. (1981). Prognostic factors in metastatic breast cancer. *Cancer Clin. Trials*, **4**, 237–247.

HAYWARD JL, CARBONE PP AND HEUSON JC. (1977). Assessment of response to therapy in advanced breast cancer. *Cancer*, **39**, 1289.

HOEKMAN K, WAGSTAFF J, VAN GC, VERMORKEN JB, BOVEN E AND PINEDO HM. (1991). Effects of recombinant human granulocyte-macrophage colony-stimulating factor on myelosuppression induced by multiple cycles of high-dose chemotherapy in patients with advanced breast cancer. *J. Natl Cancer Inst.*, **83**, 1546–1553.

HORTOBAGYI GN, BODEY GP AND BUZDAR AU. (1987). Evaluation of high-dose versus standard FAC chemotherapy for advanced breast cancer in protected environment units: a prospective randomized study. *J. Clin. Oncol.*, **5**, 354–364.

HRYNIUK W AND BUSH H. (1984). The importance of dose intensity in chemotherapy of metastatic breast cancer. *J. Clin. Oncol.*, **2**, 1281–1288.

MICK R, BEGG CB, ANTMAN KH, KORZUN AH AND FREI EI. (1989). Diverse prognosis in metastatic breast cancer: who should be offered alternative initial therapies? *Breast Cancer Res. Treat.*, **13**, 33–38.

MILLWARD MJ, HARRIS AL AND CANTWELL B. (1990). Phase II study of doxorubicin plus ifosfamide/mesna in patients with advanced breast cancer. *Cancer*, **65**, 2421–2425.

PATERSON A, SZAFRAN O AND CORNISH F. (1981). Effect of chemotherapy on survival in metastatic breast cancer. *Breast Cancer Res. Treat.*, **1**, 357–363.

SCHOLL SM, ASSELAIN B, PALANGIE T, DORVAL T, JOUVE M, GIRALT EG, VILCOQ J, DURAND JC AND POUILLART P. (1991). Neoadjuvant chemotherapy in operable breast cancer. *Eur. J. Cancer*, **27**, 1668–1671.

TANNOCK IF, BOYD NF, DEBOER G, ERLICHMAN C, FINE S, LAROCQUE G, MAYERS D, PERRAULT D AND SUTHERLAND H. (1988). A randomized trial of two dose levels of cyclophosphamide, methotrexate, and fluorouracil chemotherapy for patients with metastatic breast cancer. *J. Clin. Oncol.*, **6**, 1377–1387.

TORMEY DC, GELMAN R AND BAND PR. (1982). Comparison of induction chemotherapies for metastatic breast cancer. An Eastern Cooperative Oncology Group trial. *Cancer*, **50**, 1235-1244.

WOOD WC, BUDMAN DR, KORZUN AH, COOPER MR, YOUNGER J, HART RD, MOORE A, ELLERTON JA, NORTON L, FERREE CR, BALLOW AC, FREI EI AND HENDERSON IC. (1994). Dose and dose intensity of adjuvant chemotherapy for stage II, node-positive breast carcinoma. *N. Engl. J. Med.*, **330**, 1253-1259.